# Terrestrial Laser Scanner Two-Face Measurements for Analyzing the Elevation-Dependent Deformation of the Onsala Space Observatory 20-m Radio Telescope’s Main Reflector in a Bundle Adjustment

**DOI:** 10.3390/s17081833

**Published:** 2017-08-09

**Authors:** Christoph Holst, David Schunck, Axel Nothnagel, Rüdiger Haas, Lars Wennerbäck, Henrik Olofsson, Roger Hammargren, Heiner Kuhlmann

**Affiliations:** 1 Institute of Geodesy and Geoinformation, University of Bonn, Bonn D-53115, Germany; s7daschu@uni-bonn.de (D.S.); nothnagel@uni-bonn.de (A.N.); heiner.kuhlmann@uni-bonn.de (H.K.); 2 Onsala Space Observatory, Chalmers University of Technology, Onsala SE-43992, Sweden; rudiger.haas@chalmers.se (R.H.); lars.wennerback@chalmers.se (L.W.); henrik.olofsson@chalmers.se (H.O.); roger.hammargren@chalmers.se (R.H.)

**Keywords:** terrestrial laser scanning, deformation analysis, parameter estimation, bundle adjustment, calibration, radio telescope, very long baseline interferometry

## Abstract

For accurate astronomic and geodetic observations based on radio telescopes, the elevation-dependent deformation of the radio telescopes’ main reflectors should be known. Terrestrial laser scanning has been used for determining the corresponding changes of focal lengths and areal reflector deformations at several occasions before. New in this publication is the situation in which we minimize systematic measurement errors by an improved measurement and data-processing concept: Sampling the main reflector in both faces of the laser scanner and calibrating the laser scanner in situ in a bundle adjustment. This concept is applied to the Onsala Space Observatory 20-m radio telescope: The focal length of the main reflector decreases by 9.6 mm from 85${}^{\circ }$ to 5${}^{\circ }$ elevation angle. Further local deformations of the main reflector are not detected.

## 1. Motivation

Terrestrial laser scanners are now widely used in engineering geodesy both for documentation and for deformation monitoring. Deformation analyses of, e.g., cooling towers [1], tunnels [2], dams [3,4] or operas [5], are tasks where the high sampling density of laser scanners is used to obtain geometric information of the scanned object nearly continuously in space. Along with this goes the desire to increase the accuracy of the derived point cloud and the results of deformation analysis. A large part of the corresponding error budget is due to the laser scanner misalignment [6]. To reduce these systematic errors, laser scanners are calibrated previous to the deformation analysis [7,8] or in situ at the same time of deformation analysis [9,10]. Herein, the benefit of using two-face measurements has been documented by [11,12] and adopted by [13].

Terrestrial laser scanning has also been used for determining changes of focal lengths and areal reflector deformations of radio telescopes at several occasions before [14,15,16,17,18,19]. New in this publication is the situation that we adopt and enhance the findings of laser scanner calibration to the deformation analysis of radio telescopes’ main reflectors. Thus, we minimize systematic measurement errors by an improved measurement concept, i.e., the scanning in two cycles or two faces, respectively. This doubled information is taken advantage of in a bundle adjustment also estimating one set of calibration parameters.

**Figure 1:**
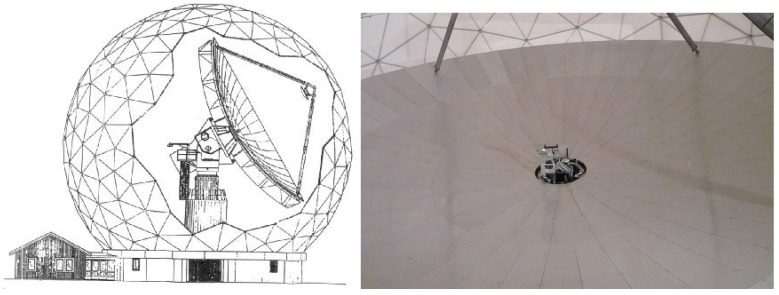
(**Left**) Sketch of the Onsala 20 m radio telescope inside the surrounding radome; (**Right**) Section of the main reflector’s surface consisting of 120 panels.

The Onsala Space Observatory (OSO) 20-m radio telescope is a mm- and cm-wave antenna with a focal length of about 9 m, located about 50 km south of Goteborg, Sweden (Figure 1 left). It consists of 120 solid panels forming the shape of a rotational paraboloid. The panels’ lengths vary between about 3.4 and 3.0 m, their widths vary between about 0.2 and 1.3 m. Their surface accuracy is of 0.14 mm to 0.22 mm (Figure 1 right). The telescope was built in 1975 and it is operated by the Swedish National Facility for Radio Astronomy, Onsala Space Observatory at Chalmers University of Technology. It is used as a single–dish instrument for astronomical measurements. It is also used for geodetic observations taking part in global VLBI (Very Long Baseline Interferometry) networks [20]. The radio telescope is enclosed by a radome (Figure 1) to avoid influences by sun or wind during its observations.

VLBI measurements employ pairs of radio telescopes to simultaneously observe signals from quasi stellar objects for determining the difference in signal arrival times. The parameters of the baseline between the radio telescopes can then be estimated from the time delay which also depends on the signal paths within the optics of the radio telescopes [21]. Hence, changes in focal length of the main reflector or local deformations on the reflector’s surface directly lead to biased signal paths and, thus, biased baseline estimates [22]. Consequently, both geometric properties should be known very accurately. As they might vary due to gravitation, they should be estimated w.r.t. the elevation angle of the radio telescope leading to an elevation-dependent deformation. The global deformation is given by the focal length variations, local deformations are expressed by deviations from the best-fit rotational paraboloid. These local deformations might result from single de-oriented panels or from adverse forces acting on the support structure of the reflector.

The OSO 20-m telescope’s main reflector has not yet been analyzed for surface deformations. To increase the accuracy of measurements performed by the OSO 20-m radio telescope, the present study, hence, proposes a new concept for an elevation-dependent deformation analysis. Herein, three main points are focused:

- Scanning the main reflector in two opposite cycles so that each part of the main reflector is sampled in two faces (Section 2).
- Proving the applicability of TLS (Terrestrial Laser Scanner) for elevation-dependent deformation analyses of radio telescopes by investigating the measurement’s accuracy in detail (Section 3).
- Analyzing the elevation-dependent deformation considering relevant systematic measurement errors based on an improved measurement and data-processing concept in a bundle adjustment (Section 4).

In consequence, this study proposes several general insights in the deformation analysis of large civil infrastructures as well as in the quality assurance when using TLS for such engineering tasks demanding high accuracy.

## 2. Measurement Concept

For the present application, a Leica Scan Station P20 laser scanner was used. In general, a TLS samples the surrounding surface by a large number *m* of scan points that are based on measuring horizontal angles $\varphi_{j}$, vertical angles $\theta_{j}$ and slope distances $r_{j}$ leading to cartesian coordinates

(1) $$x_{j}=\left[\begin{array}{c} x_{j}\\ y_{j}\\ z_{j} \end{array}\right]=\left[\begin{array}{c} r_{j}\times \sin\theta_{j}\times \sin\varphi_{j}\\ r_{j}\times \sin\theta_{j}\times \cos\varphi_{j}\\ r_{j}\times \cos\theta_{j} \end{array}\right]$$

where j=1,…,m. Here, the horizontal and vertical sampling follows a constant grid, i.e., $\theta_{j}=\theta_{1}+j\times \Delta \theta$ and $\varphi_{j}=\varphi_{1}+j\times \Delta \varphi$ with Δθ=Δφ being the angle increments. The distances are gained by measuring the time-of-flight of the emitted laser pulse reflected at the object. The horizontal rotation of the instrument is usually restricted to 180${}^{\circ }$ only in one single scan cycle since the laser scanner measures in face 1 (in front of the scanner) as well as in face 2 (behind the scanner). By this common procedure, measuring time is saved.

**Figure 2:**
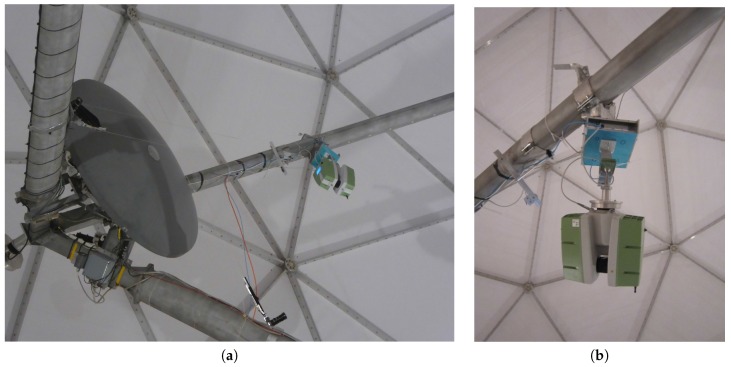
(**a**) Mounting of the laser scanner near the subreflector at one of the radio telescope’s beams by a home-made support; (**b**) Detail view of laser scanner and spherical hinge.

For analyzing the deformation and movement of the radio telescope’s main reflector, the scans were performed at different elevation angles of the telescope: 85${}^{\circ }$, 75${}^{\circ }$, 60${}^{\circ }$, 45${}^{\circ }$, 30${}^{\circ }$, 15${}^{\circ }$ and 5${}^{\circ }$. At each of these elevation angles, the main reflector should be densely sampled by a point cloud without large occlusions. This can only be achieved if the scanner is mounted on the radio telescope itself so that it moves together with the main reflector when changing the elevation angle. Similar to previous studies at the Effelsberg 100 m radio telescope [18], positioning the laser scanner at the subreflector would have been preferable. However, since the Onsala 20 m radio telescope is of smaller size, a station could only be realized by a support that was fixed to one of the four beams holding the subreflector (Figure 2). As a consequence, the support legs produce occlusions. In addition, the main reflector could not be scanned completely at elevation angles of 15${}^{\circ }$ and 5${}^{\circ }$ due to the limited vertical field-of-view of 270${}^{\circ }$ of the TLS. However, this concerns only a small part of the reflector’s surface.

The support holding the TLS has been designed and manufactured particularly for TLS instruments scanning radio telescopes. It consists of a bracket being attached to the radio telescope’s beam and a flexible spherical hinge (Figure 2). The flexible spherical hinge was used so that the laser scanner is always oriented upside-down, independent of the telescope’s elevation angle.

During the rotation of the telescope, the hinge was unconstrained while during measurements, a pneumatic break held the scanner in a vertical position. The assumption was that the scanner stays always upside-down due to its weight. Only after the fixation of the hinge, the scanner started its own rotation and measurements assuming a stable position and orientation. As will be analyzed in Section 3, this assumption of a stable laser scanner station is only partially valid.

The following scan settings were chosen for scanning at the proposed elevation angles:

- Spatial resolution: 1.6 mm at 10 m distance. This high point sampling is not necessary for the parameter estimation but it helps at determining spatial correlations in laser scans which is the task of a further study.
- Spatial coverage: Complete scan of 360${}^{\circ }$ horizontal and 270${}^{\circ }$ vertical coverage (full vertical field-of-view).
- Quality level: The Leica Scan Station P20 requires the user to specify a quality level. The larger the quality level, the more points being consecutively measured are averaged to minimize random errors in the measured distances. This moving average most probably leads to a larger correlation between the scan points with unknown error distribution that would have to be accounted for in the deformation analysis. Hence, although a high quality level would indeed increase the precision of the distance measurement, the lowest quality level (level “1”, no averaging) was chosen.

Special focus should be led on the horizontal rotation of the laser scanner during a complete scan: Since the area of interest covers more than 180${}^{\circ }$ in the horizontal direction, scanning only in one cycle which equals a horizontal rotation of 180${}^{\circ }$—as is the usual procedure at laser scanning—would lead to that some parts of the main reflector were scanned solely in face 1 and other parts are scanned solely in face 2. This would degrade the accuracy of the deformation analysis due to systematic errors resulting from the laser scanner misalignment [11,12]. Consequently, all scans were performed in both faces by instructing the laser scanner to horizontally rotate from 0${}^{\circ }$ to 180${}^{\circ }$ (cycle 1) as well as from 180${}^{\circ }$ to 360${}^{\circ }$ (cycle 2). In consequence, each part of the reflector is scanned in face 1 as well as in face 2 which is taken advantage of in the deformation analysis (Section 4).

**Figure 3:**
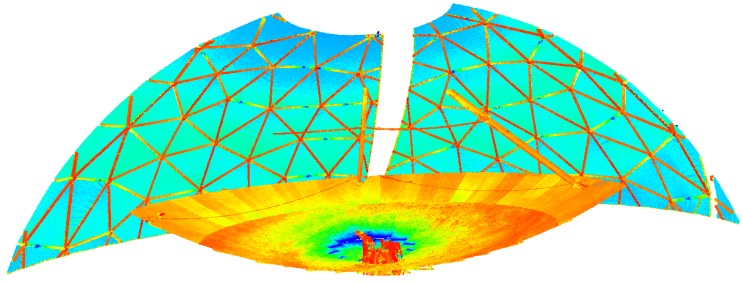
Section of the point cloud with intensity-based coloring acquired at an elevation angle of 85${}^{\circ }$ in cycle 1: blue represents a high intensity, followed by green, red represents a low intensity.

These settings led to 14 scans in total, each of the seven elevation angles measured in two cycles. Each point cloud consists of about 570 million points. Figure 3 exemplarily depicts the intensity coded point cloud of elevation angle 85${}^{\circ }$ in cycle 1: The main reflector is visible in the foreground by a rather low intensity, the radome is visible in the background with different intensities of the points lying on the metal beams and its hull.

## 3. Preliminary Accuracy Evaluation of Measurements

Before performing the deformation analysis in Section 4, the accuracy of the laser scans is evaluated. In general, this accuracy is governed by random and systematic errors being due to the laser scanner’s misalignment, atmospheric conditions, the scanning geometry and the object properties [23]. Here, systematic errors are often larger than the random ones. Concerning the measurements on the main reflector, the following statements can be formulated:

1. The atmospheric conditions did not vary noticeably with time during the measurements due to cloudiness: The absolute air temperature was 14.1${}^{\circ }$ Celsius on average, the variation smaller than 0.2${}^{\circ }$ Celsius. Since the measured distances are only between 7 and 13 m, systematic errors in the measured distances due to atmosphere can, thus, be neglected regarding the total error budget of laser scanners.
2. The scanning geometry is chosen considering angles of incidence of up to 45${}^{\circ }$. Thus, systematic errors in the measured distance due to large angles of incidence, as investigated by [24], can be neglected.
3. The panels of the main reflector are bare aluminium with a light gray color but manufactured very smoothly to receive radio signals of small wavelengths. Hence, the intensity of the reflected laser spot and the random errors that are governed by this intensity [25] have to be investigated further (see Section 3.3).
4. Systematic errors due to laser scanner misalignment have already been proven to be a limiting factor for deformation analyses. Consequently, they also have to be investigated further (see Section 3.2).

In the present study, the stability of the laser scanner is also of great importance: Contrary to the usual mounting of a laser scanner on a tripod or a pillar, the laser scanner is fixed to a flexible spherical hinge whose stability has not yet been proven. This is investigated in Section 3.1.

For the subsequent evaluations of the laser scans’ uncertainty, the laser scans are approximated by a rotational paraboloid representing the main reflector of the radio telescope. This procedure—which is only used in this section to investigate the derived residuals of approximation—will be explained in more detail in Section 4.

### 3.1. Stability of Laser Scanner Mounting

The stability of the laser scanner is of particular interest here since its mounting is rather unusual. This is due to two facts: (1) The laser scanner is oriented always upside-down and (2) it is mounted neither on a tripod nor a concrete pillar but to a flexible spherical hinge which is moving itself together with the main reflector.

**Figure 4:**
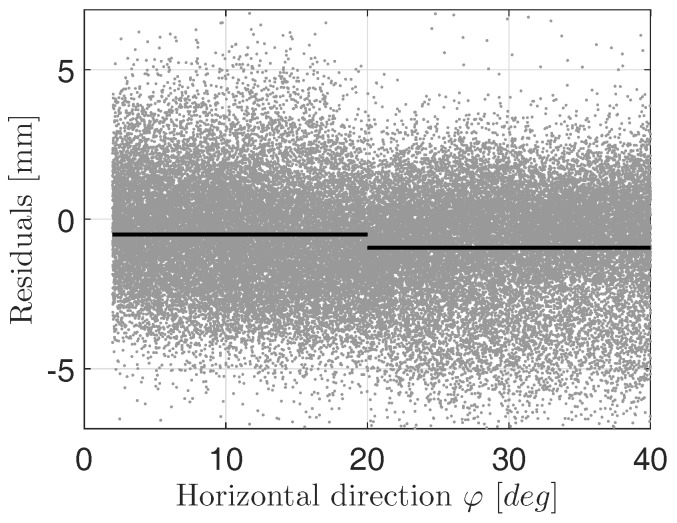
Residuals of approximation at an elevation angle of 60${}^{\circ }$, cycle 1 (grey); averaged residuals before and after 20${}^{\circ }$ horizontal direction (black line).

Figure 4 depicts a section of the residuals of adjustment when approximating a rotational paraboloid to the point cloud of the main reflector at an elevation angle of 60${}^{\circ }$, only using cycle 1. The residuals are sorted by the measured horizontal direction where a direction of 0${}^{\circ }$ represents the start of measurement. It can be seen that there is an abrupt shift between the residuals before and after horizontal directions of 20${}^{\circ }$. This bias is highlighted by calculating the average of all residuals before and after 20${}^{\circ }$ (black line). A similar behavior can be seen for other cycle 1 scans: Elevation angles of 85${}^{\circ }$ and 75${}^{\circ }$. At cycle 2 scans, this bias does not exist.

The amount, as well as the orientation where the shift occurs, varies between the individual scans. Thus, the position of this shift on the main reflector also varies. Hence, we cannot claim the main reflector’s surface to produce this bias by an abrupt local deformation. The reason for this bias has rather to be searched in the data acquisition. This assumption can be supported by the fact that the bias only occurs at some of the cycle 1 scans.

Instead, the bias can be interpreted as a poor stability of the laser scanner orientation: It only occurs at measurements in cycle 1. These scans were performed directly after the movement of the main reflector around its elevation axis. Hence, when the scanner started its rotations around its vertical axis, the spherical hinge supporting the laser scanner might not have been fixed in its correct position so that the rotation of the scanner forced the hinge to re-orientate until it was in its desired upside-down position. After this re-orientation, the hinge was apparently always stable enough since this shift only occurs in cycle 1 measurements and only at the beginning of these scans.

In contrast to this, at elevation angles of 5${}^{\circ }$, 15${}^{\circ }$, 30${}^{\circ }$ and 45${}^{\circ }$, the shift is not visible. Hence, the orientation of the scanner seems to have been upside-down directly after movement of the telescope in these cases. In consequence, the scans of elevation angle 85${}^{\circ }$, 75${}^{\circ }$ and 60${}^{\circ }$ of cycle 1 suffer from systematic errors due to the instability of the laser scanner mount. These point clouds are, thus, not used in the further analyses of this study.

### 3.2. Systematic Errors Due to Misalignment of the TLS

**Figure 5:**
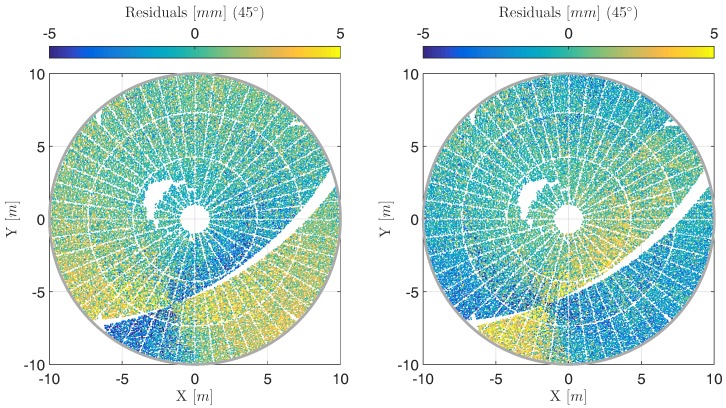
(**Left**) Residuals of the deformation analysis at an elevation angle of 45${}^{\circ }$ at cycle 1 shown in object coordinate system (X,Y,Z) where the *X*-axis equals the tilting axis; (**Right**) Corresponding residuals at cycle 2; The reasons for the three gaps in the point cloud are explained in Section 4.

Systematic errors following from unavoidable misalignment of the TLS can easily outnumber the random errors so that the interpretation of, e.g., deformations, can be totally misleading. This can also be seen in the present study: Figure 5 displays the residuals of approximation for cycle 1 (left) and cycle 2 (right). It is visible that the residuals are systematically distributed with opposite sign between cycle 1 and cycle 2. Therefore, the sign also changes between face 1 and face 2 measurements of the scanner. This implies that these systematics are not due to the deformation of the main reflector but due to the misalignment of the laser scanner being sensitive to two-face measurements for a large amount [13]. Consequently, the laser scanner needs to be calibrated for a meaningful deformation and movement analysis.

The aim of the laser scanner calibration is the parameterization of the laser scanner’s misalignment which seems to be similar to the calibration of a total station at first glance. Hence, calibration parameters as, e.g., collimation axis error, trunnion axis error and vertical index offset need to be determined. However, the calibration of a terrestrial laser scanner is more complex [6], mainly due to three reasons:

- The observations of the TLS are pre-processed by the manufacturer before made available for the operator. Here, the original measurements are corrected by unknown calibration functions. The calibration parameters used herein are determined during manufacturing; they vary with temperature. Consequently, the remaining errors due to misalignment that we want to estimate here are temperature-dependent and do only represent rest effects that are not handled by the manufacturer’s pre-calibration.
- Since the mechanical construction of a TLS is complex, there are still different opinions about which calibration parameters to estimate. On the one hand, there are empirical parameters [8,26], on the other hand, there are mechanical parameters introduced by [11] and adopted by [13] who coincide to a large amount, but not in total. With both strategies, systematic errors can be reduced but the parameter estimates vary.
- You cannot measure single scan points repeatedly so that a sophisticated network of reference objects has to be build up for estimating the calibration parameters. Only at optimal networks, the calibration parameters are assumed to be stable.

In consequence, the present strategies for laser scanner calibration still suffer from the fact that the calibration parameters are only valid in situ [7,8,27,28,29,30]. This means that they are only valid for the measurement setup used for calibration and that they might change when using a different strategy for calibration. Strategies to overcome this drawback are given by, e.g., [13]. Anyway, since there is currently no strategy at hand for calibrating a laser scanner so that only random errors would remain in the point clouds acquired in the present study, the deformation analysis shown here has to handle these systematic errors due to misalignment.

Hence, the parameters describing the potential mechanical misalignment of the laser scanner are estimated as part of the common deformation analysis leading to an in situ self-calibration. This approach has been introduced for the deformation analysis of the Effelsberg 100-m radio telescope’s main reflector by [9,18] including also a sensitivity analysis. However, deviating from the present study, the Effelsberg radio telescope was not scanned in two cycles or faces, respectively. Furthermore, the functional model for parameterizing the laser scanner’s misalignment has been improved meanwhile by [11] by introducing mechanically reasonable parameters.

Due to the laser scanner misalignment, the calculation of the cartesian coordinates written in Equation (1) has to be expanded by a set of calibration parameters:

(2) $$\Delta \varphi_{j}=\frac{x_{1z}}{r_{j}\tan\theta_{j}}+\frac{x_{3}}{r_{j}\sin\theta_{j}}+\frac{x_{5z-7}}{\tan\theta_{j}}+\frac{2x_{6}}{\sin\theta_{j}}$$

(3) $$\Delta \theta_{j}=\frac{x_{1n+2}\cos\theta_{j}}{r_{j}}+x_{4}+x_{5n}\cos\theta_{j}-\frac{x_{1z}\sin\theta_{j}}{r_{j}}$$

Consequently, $\varphi_{j}+\Delta \varphi_{j}$ and $\theta_{j}+\Delta \theta_{j}$ are integrated in Equation (1) instead of solely $\varphi_{j}$ and $\theta_{j}$ considering the calibration parameters $p_{calib}$ (calib = calibration)

(4) $$p_{calib}={\left[x_{1z},x_{3},x_{5z-7},x_{6},x_{1n+2},x_{4},x_{5n}\right]}^{T}.$$

All of these parameters are two-face sensitive; hence, they change their signs between face 1 and face 2. To account for this, the angle parameterization is adapted following [13]: In the second cycle, the value of 180${}^{\circ }$ is added to the horizontal angle if the calculated angle is positive and 360${}^{\circ }$ if the angle is negative. The position of the mirror is easily tracked with the $x_{j}$-coordinate value. If this coordinate in the first cycle is negative, the calculated vertical angle is subtracted from 360${}^{\circ }$. In the second cycle, the case is opposite: If the $x_{j}$-coordinate is positive, the calculated vertical angle is subtracted from 360${}^{\circ }$.

In general, a panoramic laser scanner is subject to 18 calibration parameters [11]. This list was adapted to the system calibration of the laser scanner used here [13], the Leica ScanStation P20, leading to 11 parameters. This number is further reduced here to 7 parameters since the other four parameters cannot be estimated based on the given measuerement configuration. In consequence, only angular calibration parameters are incorporated in Equations (2) and (3): Vertical beam offset $x_{1z}$, mirror offset $x_{3}$, trunnion axis error $x_{5z-7}$, collimation axis error $x_{6}$, horizontal beam offset $x_{1n+2}$, vertical index offset $x_{4}$ and horizontal beam tilt $x_{5n}$.

**Figure 6:**
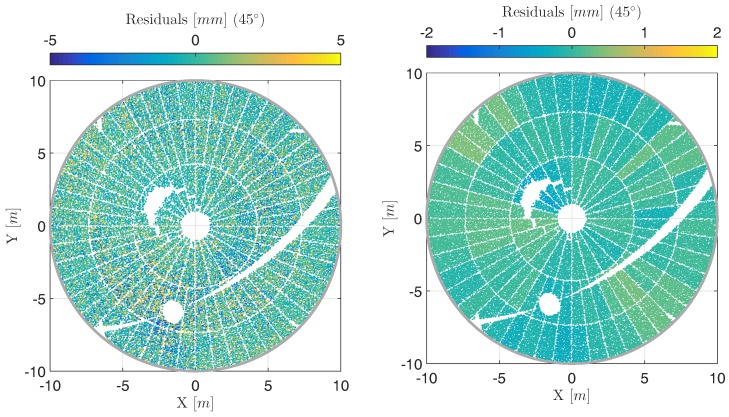
(**Left**) Residuals of deformation analysis when estimating calibration parameters at an elevation angle of 45${}^{\circ }$ at cycle 1 shown in object coordinate system (X,Y,Z) where the *X*-axis equals the tilting axis; (**Right**) Corresponding panel-wise averaged residuals according to Section 3.3 (notice different color scale).

Figure 6 (left) depicts the residuals at an elevation angle of 45${}^{\circ }$, cycle 1, if approximating the point cloud by a rotational paraboloid according to the procedure that will be introduced in Section 4. This strategy accounts for the calibration parameters of Equation (4) by an in situ calibration using a bundle adjustment. Compared to Figure 5 (left) that shows the results without calibration, the benefit is obvious: The systematic of opposite sign is eliminated increasing the accuracy of the angular measurements significantly. The remaining effects on the surface are interpreted together with the results corresponding to the other elevation angles in the end of this study.

In consequence, any deformation analysis should be accompanied by an in situ self-calibration of the laser scanner. By this, the accuracy of the measured horizontal angles $\varphi_{j}$ and vertical angles $\theta_{j}$ is increased leading to an improved deformation analysis. This aspect will be included in Section 4.

It should be noted that the estimation of the calibration parameters goes along with an elimination of a small part of the scan points as can be seen in, e.g., Figure 6:

- The calibration parameters $x_{1z}$ and $x_{5z-7}$ influence the horizontal direction $\varphi_{j}$ by a factor of $\frac{1}{\tan\theta_{j}}$. This factor is not well defined at zenith and its close proximity. Hence, scan points with $|\theta |<5^{\circ }$ are eliminated leading to a circular gap in the point cloud.
- The scanner does not rotate exactly 180${}^{\circ }$ around its vertical axis but 184${}^{\circ }$ for each cycle, e.g., from −2${}^{\circ }$≤φ≤182${}^{\circ }$. Consequently, there are always 4${}^{\circ }$ of overlap between cycle 1 and cycle 2 or face 1 and face 2, respectively. The scan points in these overlaps cannot be assigned to face 1 or face 2. Hence, the corresponding scan points are eliminated leading to two further gaps of elongated form.

### 3.3. Precision of Distance Measurements

**Figure 7:**
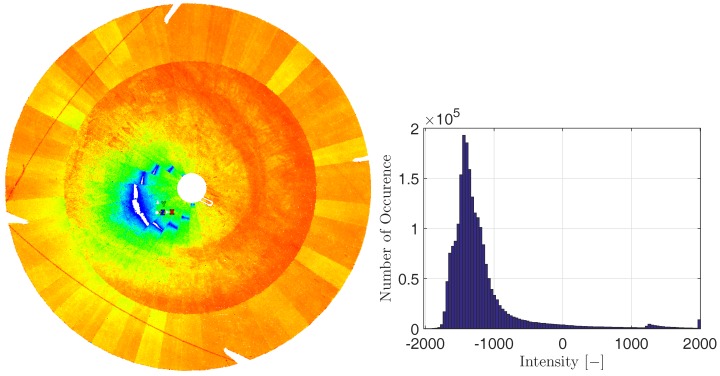
(**Left**) Relative intensity (unit-less) of the scan points on the main reflector at an elevation angle of 85${}^{\circ }$ in cycle 1; blue represents a high intensity, followed by green, red represents a low intensity; (**Right**) Histogram of intensity values.

The intensity of the backscattered laser spot governs the precision of the distance measurement [25]. While Figure 3 already depicted this intensity for the section of one scan, Figure 7 (left) focuses the intensity of the scan points on the main reflector. As can be seen in the latter case, the intensity partially depends on the angle of incidence:

- The positions where the intensity is very high (dark blue), the angle of incidence is about 5${}^{\circ }$ or even less. In the vicinity of angle of incidences around 0${}^{\circ }$, the reflected intensity is so high that the reflected impulse cannot be processed by the scanner due to blooming. This leads to gaps in the point cloud. The sensitivity of this blooming regarding the angle of incidence can be seen since the position of theses gaps changes between different panels of the main reflector.
- At angles of incidence from 5${}^{\circ }$ to about 10${}^{\circ }$, the intensity decreases slightly (colored from light blue to green).
- At angles of incidence larger than about 10${}^{\circ }$ to the maximum values of about 45${}^{\circ }$, the intensity is not governed by the incidence angle anymore (colored from yellow to red). Here, rather the surface properties govern the intensity since individual panels can be distinguished for the intensity values.

The actual intensity values that are given by the software Cyclone as relative, unit-less numbers between −2000 and 2000 are depicted in Figure 7 (right). This histogram confirms the previous statements: Only a small portion of scan points is of large intensity (>0) due to angles of incidence smaller than about 5${}^{\circ }$. The predominant part of the scan points is of lower intensity in the range of −1800 to −800. Consequently, since the intensity values can be used to assess the precision of the distance measurements [25], this precision can be assumed to be quite constant over the whole main reflector. Excluded from this presumption are only the sections sampled by small angles of incidence.

For empirically assessing the precision of the distance measurement, the standard deviation of the estimated residuals is used: This standard deviation represents the precision of the distance measurement since (1) the residuals approximately point into the line-of-sight of the laser scanner and (2) the panels are manufactured more precisely than the range noise: The surface accuracy of the panels is specified as 0.14 to 0.22 mm [20].

**Figure 8:**
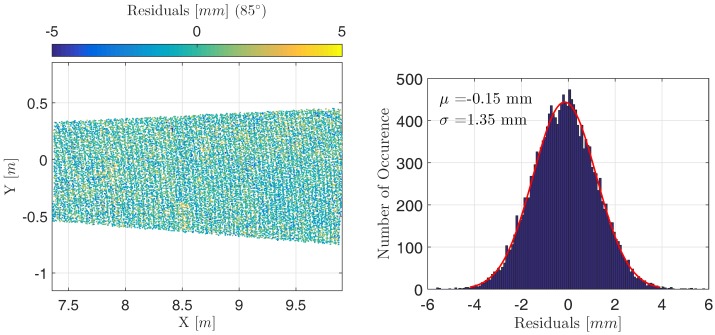
(**Left**) Residuals of deformation analysis for one single panel; (**Right**) Corresponding histogram.

Figure 8 (left) depicts one panel and its estimated residuals in detail, Figure 8 (right) the corresponding histogram. It can be seen that the residuals vary randomly on the panel’s surface and that the corresponding error distribution equals a Gaussian distribution with parameters μ = 0.15 mm and σ = 1.35 mm. While the mean values μ of all panels range between −1 and 1 mm, the standard deviation σ does not vary considerably. This statement even holds for the panels sampled by small angles of incidence whose intensity is higher.

As a consequence of these investigations, the residuals of approximation are panel-wise averaged in the subsequent deformation analysis: For each of the 120 panels, the scan points sampling it are selected and the mean value of all corresponding residuals is calculated. This averaging helps to reveal local surface deformations by increasing the signal-to-noise ratio: It minimizes random errors and it highlights systematic errors caused by local surface deformations. The result of this panel-wise averaging is shown in Figure 6 (right) in comparison to the result without panel-wise averaging in Figure 6 (left).

## 4. Analysis of Elevation-Dependent Deformations

One of the main aspects of the present study is the elevation-dependent deformation analysis of the main reflector of the OSO 20-m radio telescope. As stated in Section 1, the most relevant geometric properties of the main reflector are its focal length and the occurrence of local deformations from the assumed shape, i.e., a rotational paraboloid. To analyze these properties, the laser scan’s of the main reflector are approximated by a rotational paraboloid. This section describes the corresponding parameterization as well as the succeeding parameter estimation which will be performed in a bundle adjustment integrating all scans of the different elevation angles. Before, the point clouds need to be pre-processed based on the findings gained in Section 3.

### 4.1. Data Pre-Processing

The data pre-processing includes the steps of data reduction, object segmentation and outlier removing that have been introduced for an accurate deformation analysis of radio telescopes [18]. To avoid repetitions, the necessity and implementation of these steps is only briefly recapitulated here.

#### 4.1.1. Data Reduction

Data reduction might be a usual step in TLS point cloud pre-processing—but only aiming at improving the handling of the data memory and processing time [31,32,33]. Only in the recent past, data reduction has been analyzed regarding its potential for upgrading the unbiasedness at area-based surface analyses [18,34]. These studies show that the sampling density should be regular to gain accurate parameter estimates, especially at deformation analyses. Since the laser scanner does not sample the surface with a regular point distance between the cartesian coordinates, the point cloud should be reduced so that the point density is nearly constant over the whole area of interest. Hence, points are eliminated. Consequently, the point clouds of about 570 million points each are reduced to about 2 million points.

#### 4.1.2. Object Segmentation

Generally, object segmentation describes the separation of the object of interest from the surrounding background not being of interest. Therefore, the method of separation is application-specific in most cases. This can range from semantic segmentation [35] to geometric segmentation [36] for, e.g., plant analysis.

In the present case, the object segmentation directly evolves out of the construction of the main reflector: The main reflector does not equal a closed body but consists of 120 solid panels (see Figure 1). The distance between neighbored panels equals several millimeters. Hence, scan points in these gaps or in their proximity will suffer from systematic errors leading to mixed pixels due to a laser spot size of about 5 mm at 10 m distance [37]. To avoid an influence of these mixed pixels on the deformation analysis, only points are considered further that are located without any doubt on a surface panel. This is guaranteed by omitting all points between the panels as well as the ones with a distance less than 50 mm to the panels’ borders.

#### 4.1.3. Outlier Removing

Outliers can degrade the deformation analysis by either biasing the parameter estimation or by complicating the analysis of local deformations on the main reflector’s surface. Hence, they need to be eliminated. In total, these outliers occur because of different reasons:

- The vertex of the main reflector: Here, the feedhorn collecting the electro-magnetic signals and further processing units are located. Hence, points inside a radius of 1 m around the vertex are removed.
- Gaps between the panels: These outliers have already been eliminated by the object segmentation.
- Blooming effects that occur at small angles of incidence (see also Section 3): The intensity given by the laser scanner ranges between a unit-less value of −2000 and +2000. An empirical threshold of 1500 is used so that points with larger intensity values are eliminated. This leads to a further small gap in the point clouds near the main reflector’s vertex.
- Common gross errors in the distance measurement that are, amongst others, due to the electronic processing or small anomalies in the surface’s reflectivity: These outliers are removed in a pre-adjustment eliminating points with estimated absolute residuals larger than 7 mm (empirically determined).

### 4.2. Parameterization of Laser Scans in Each Elevation Angle

The measured point clouds of the main reflector each consist of j=1,…,m sampling points that can be parameterized by the functional model of rotational paraboloid. A rotational paraboloid can be described by one form parameter only, i.e., the focal length *f*, if it is positioned in its normal form where its rotation axis equals the *Z*-axis of the coordinate system (X,Y,Z):

(5) $$\begin{array}{c} \frac{X_{j}^{2}+Y_{j}^{2}}{4\times f}-Z_{j}=0. \end{array}$$

This is, however, not the case since the main reflector is scanned in the laser scanner’s local coordinate system (x,y,z), so that the paraboloid needs to be transformed by

(6) $$\begin{array}{c} X_{j}=\left[\begin{array}{c} X_{j}\\ Y_{j}\\ Z_{j} \end{array}\right]=R_{y}\left(\phi_{y}\right)\times R_{x}\left(\phi_{x}\right)\times x_{j}+X_{v} \end{array}$$

where $R_{x}$ and $R_{y}$ are the rotation matrices around the *x*- and *y*- axis and $X_{v}$ is the translation vector. Consequently, the parameters $p_{obj}$ (obj = object) describing the rotational paraboloid in the laser scanner’s coordinate system equal

(7) $$\begin{array}{c} p_{obj}={\left[X_{v},Y_{v},Z_{v},\phi_{x},\phi_{y},f\right]}^{T}. \end{array}$$

For estimating the parameters $p_{obj}$ (Equation (7)), the original polar measurements of the TLS $r_{j}$, $\varphi_{j}$ and $\theta_{j}$ are integrated in the paraboloid’s model (Equation (5)) by transformation into cartesian coordinates $x_{j}$ (Equation (1)) and then by transformation into the coordinates $X_{j}$ of the paraboloid’s coordinate system (Equation (6)).

### 4.3. Bundle Adjustment of All Laser Scans

Equations (5)–(7) introduced the parameterization of the main reflector. If performing a least-squares parameter estimation based on these equations, only random errors are dealt with. However, as has been explained in Section 3, also systematic errors due to misalignment of the laser scanner are included in the point clouds. Thus, we need to find a strategy to minimize these systematic errors.

In principle, there could be two strategies:

1. Simply measuring the object in two faces: By combining both point clouds, all two-face sensitive errors can be minimized.
2. Calibrating the laser scanner in situ: The calibration parameters are estimated along with the deformation parameters.

We focus a combination of both strategies for the present application. This can be reasoned by the following: At the elevation angles 85${}^{\circ }$, 75${}^{\circ }$ and 60${}^{\circ }$, the measurements for cycle 1 cannot be used for analysis due to the instability of the spherical hinge. Hence, at these elevation angles, we only can use point clouds that sampled each part of the main reflector with one single face. Strategy 1 is, thus, not feasible here. Hence, an in situ calibration as has been done many times before—also for radio telescopes as described in Section 3.2—is necessary in any case. This in situ calibration benefits if also two-face measurements are included corresponding to [12,13] since most calibration parameters are two-face sensitive [11].

Consequently, we estimate the 7 calibration parameters $p_{calib}$ describing the laser scanner misalignment along with the object parameters $p_{obj}$ to account for systematic measurement errors. These 7 calibration parameters are specified in Equations (2) and (3); only two-face sensitive parameters are used. We only use the 7 two-face sensitive parameters since the configuration of adjustment is not sensitive to estimate the other 4 calibration parameters. In an ideal case, all 11 parameters would have been estimated. Since this is not the case here, the remaining errors due to the 4 non-estimated parameters—that is not dealt with—merge with the calibration parameters that are estimated. This interdependency has already been discussed in [9] and it will be addressed in a prospective study where we also compare the different strategies for dealing with the systematic laser scanner errors due to misalignment (two-face measurements, in situ calibration, combination of both) extensively.

While the calibration parameters $p_{calib}$ are expected to be constant for each laser scanner—or they are at least expected to stay constant at similar temperatures and for a longer period than one day—the parameters $p_{obj}$ vary with the elevation angle *e*: $p_{obj}\left(e\right)$. Therefore, the laser scans of the different elevation angles can be linked in a bundle adjustment since they share identical calibration parameters. Consequently, the parameters to be estimated in one single bundle adjustment equal

(8) $$p=\left[\begin{array}{c} p_{obj}\left(85^{\circ }\right)\\ \vdots \\ p_{obj}\left(5^{\circ }\right)\\ p_{calib} \end{array}\right]$$

leading to u=49 parameters, i.e., 42 object parameters and 7 calibration parameters. The number of observations *n* equals n=3×m where the number *m* of scan points is about 22 million. This is because 7 elevation angles were scanned in two cycles but the scans of cycle 1 at elevation angles 85${}^{\circ }$, 75${}^{\circ }$ and 60${}^{\circ }$ are disregarded for deformation analysis due to missing stability of the laser scanner (Section 3). To improve the handling of this large number of scan points, it is further reduced to m≈ 4 million points. Still, the redundancy of the adjustment is very large: f=m−u≈m=4 million.

For the stochastic model, the covariance matrix

(9) $$\begin{array}{c} \Sigma_{ll}=\left[\begin{array}{cccc} \sigma_{r}^{2} & & & \\ & \sigma_{\varphi }^{2} & & \\ & & \sigma_{\theta }^{2} & \\ & & & \ddots \end{array}\right] \end{array}$$

of size n×n is formulated where $\sigma_{\varphi }=8^{\prime \prime }$ and $\sigma_{\theta }=8^{\prime \prime }$ are chosen corresponding to the manufacturer’s specifications of the Leica Scan Station P20 [37]. For the distance $r_{j}$, a standard deviation of $\sigma_{r}=1.5$ mm is chosen due to the evaluations of Section 3. This value only slightly differs from the manufacturer’s specifications that do not account for the present object properties. As usual, the observations are assumed to be uncorrelated and Gaußian distributed, which is—in general—an oversimplification of the reality [19,38,39].

This adjustment typically leads to the Gauß-Helmert model (GHM)—also known as general case of adjustment [40]—considering the used functional model. Thus, the residuals $\hat{v}$ representing the deviations between observations l and approximated observations $\hat{l}$ are minimized following the theoretical target function $v^{T}\Sigma_{ll}^{-1}v$ [40,41]. The strict solution of the GHM is explained in many publications [40,42] so that a recapitulation is omitted here. Due to the large number of observations, the GHM is transformed to a Gauß-Markov model for numerical reasons [34,41].

The results of the approximation are the estimated parameters $\hat{p}$ including their covariance matrix $\Sigma_{\hat{p}\hat{p}}$. Here—as already stated—the estimated focal length is the most meaningful parameter for the present application. Furthermore, the post-fit residuals of each sampling point, also called discrepancies in a GHM, are estimated. They can be used to detect systematic effects in the residuals possibly stemming from local deformations of the telescope’s surface.

### 4.4. Results of Adjustment

The results of the adjustment can be split up into the estimated calibration parameters, the estimated object parameters indicating the global deformation and the post-fit residuals indicating local deformations. Before these are evaluated, the parameter correlations are investigated.

#### 4.4.1. Parameter Correlations

In general, parameter correlations help at investigating the ability to separate estimated parameters from each other. In the present case, two questions are of special interest:

1. Do the two-face measurements improve the separability between object and calibration parameters?
2. Does the bundle adjustment improve the separability between object and calibration parameters?

If both questions could be answered with ’yes’, the significance of the deformation analysis would increase since the values of the object parameters are less affected by the in situ calibration of the TLS.

**Figure 9:**
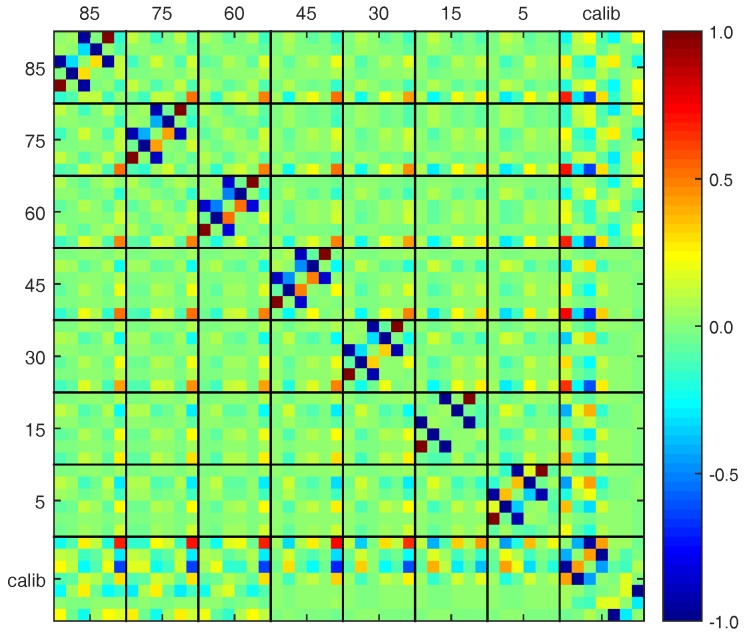
Correlation between the estimated object parameters $p_{obj}$ (85${}^{\circ }$), ..., $p_{obj}$ (5${}^{\circ }$) and the calibration parameters $p_{calib}$ at the bundle adjustment. The 6 object parameters of each elevation angle are ordered according to Equation (7), the 7 calibration parameters according to Equation (4).

Concerning the first question, we compare (a) the correlations between the object parameters $p_{obj}$ (85${}^{\circ }$), $p_{obj}$ (75${}^{\circ }$), $p_{obj}$ (60${}^{\circ }$) and the calibration parameters $p_{calib}$ (three left black boxes at bottom of Figure 9) with (b) the correlations between the object parameters $p_{obj}$ (45${}^{\circ }$), $p_{obj}$ (30${}^{\circ }$), $p_{obj}$ (15${}^{\circ }$), $p_{obj}$ (5${}^{\circ }$) and the calibration parameters $p_{calib}$ (4th to 7th black box from left at bottom of Figure 9): At (a), only one-face measurements are included due to the lack of TLS stability of the firstly scanned cycles (see Section 2). At (b), both faces are included. The correlations between $x_{1n+2}$ (5th calibration parameter) and the object parameters and also between $x_{5n}$ (7th calibration parameter) and the object parameters are noticeably larger in case (a) compared to case (b). What is not shown here: If in all elevation angles only one-face measurements had been included, the correlation between the object parameters and all calibration parameters would increase even more. Thus, including two-face measurements improves the separability of the estimated parameters.

Concerning the second question, we compare the correlations between the calibration parameters and the object parameters considering the different elevation angles (1st to 7th black box from left at bottom in Figure 9): Some correlations increase with decreasing elevation angles (e.g., the one between $Y_{v}$ and $x_{5z-7}$, 3rd calibration parameter), some decrease (e.g., the one between *f* and $x_{1z}$, 1st calibration parameter). Consequently, the combination of the different elevation angles in one bundle adjustment improves the separability of the object parameters from the calibration parameters.

#### 4.4.2. Calibration Parameters

Table 1 depicts the estimated calibration parameters. All calibration parameters are significant regarding their estimated standard deviation. Hence, w.r.t adjustment theory, they are needed for parameterizing all information included in the laser scans. However, this statement has to be softened: The estimated standard deviations are all too optimistic due to the neglect of correlations in the stochastic model which has been analyzed in detail in [38,39]. Multiplying the standard deviations by a factor of at least 10 would most probably lead to more realistic estimates. Hence, the significance of these parameters cannot be stated terminatorily here.

#### 4.4.3. Object Parameters

The estimated object parameters are presented in Table 2. Here, the focal length $\hat{f}$ and its variation $\Delta \hat{f}$ compared to the estimate at 85${}^{\circ }$ elevation are of main interest since they represent the deformation of the main reflector as a whole. All variations $\Delta \hat{f}$ are significant regarding the estimated standard deviations of maximal ${\hat{\sigma }}_{\Delta f}=\sqrt{2}\times {\hat{\sigma }}_{f}$. However, these standard deviations are—again—too optimistic. The estimated transformation parameters will be relevant when analyzing the main reflector’s movement which will be handled in a prospective study.

**Figure 10:**
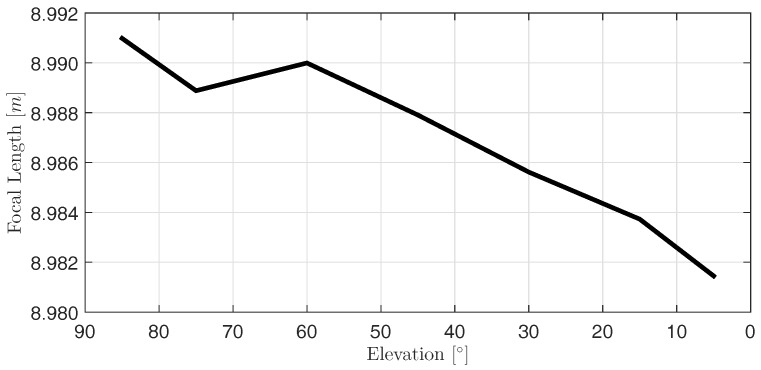
Estimated elevation-dependent variation of focal length $\hat{f}$.

Figure 10 depicts the elevation-dependent focal length of the main reflector. It decreases by 9.6 mm between 85${}^{\circ }$ and 5${}^{\circ }$. The only exception from a homogeneous decrease is the estimate at an elevation angle of 75${}^{\circ }$. This behavior seems to be unlikely. However, a metrological explanation is not at hand yet.

#### 4.4.4. Post-Fit Residuals

**Figure 11:**
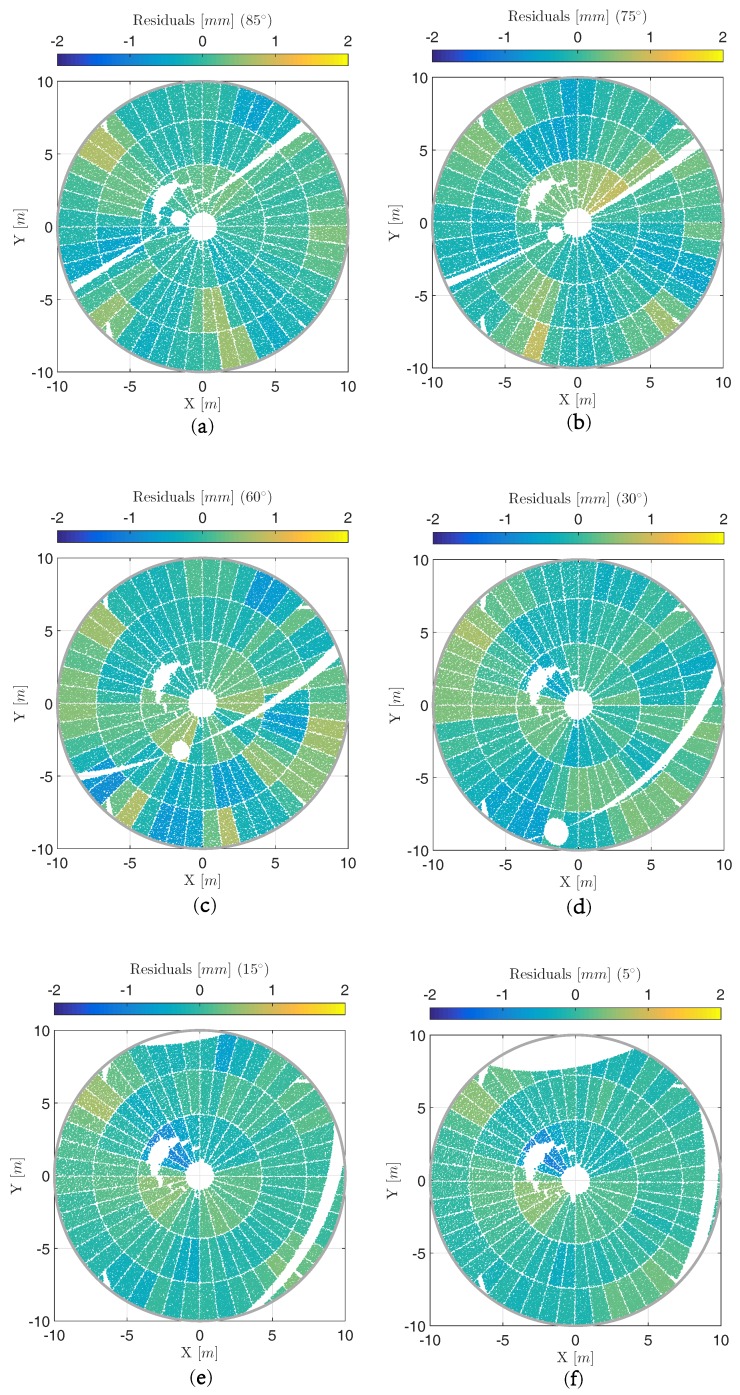
(**a**–**f**) Panel-wise averaged post-fit residuals of final deformation analysis at different elevation angles shown in each object coordinate system (X,Y,Z) where the X-axis equals the tilting axis; At elevation angles 5${}^{\circ }$ and 15${}^{\circ }$, parts of the surface cannot be scanned by the TLS due to occlusions.

The estimated residuals are relevant for analyzing local deformations of the main reflector’s surface that might concern only single panels or a collection of adjacent panels. These residuals are depicted in Figure 11 for each elevation angle. The ones for 45${}^{\circ }$ elevation angle have already been displayed in Figure 6 (right). At the elevation angles of 85${}^{\circ }$, 75${}^{\circ }$ and 60${}^{\circ }$, only one scan, i.e., cycle 1, is processed. Here, the residuals presented are the direct results of the adjustment with a subsequent panel-wise averaging of the residuals as has been introduced in Section 3.

For elevation angles of 45${}^{\circ }$, 30${}^{\circ }$, 15${}^{\circ }$ and 5${}^{\circ }$, the presented residuals are the results of an additional averaging of the residuals estimated for the laser scans of cycle 1 and cycle 2. Hence, systematic effects that have not been considered by the in situ calibration are further reduced. This increases the accuracy for determining local deformations.

The estimated residuals in Figure 6 (right) and Figure 11 do not reveal any local deformations on the main reflector’s surface. The residuals are all in the range of ± 1.5 mm which can be assumed to represent remaining random and systematic errors in the laser sans. A strict statistical evaluation of these residuals is not feasible due to the oversimplified stochastic model. This holds for all elevation angles. The fact that the magnitude of some residuals is larger at the elevation angles of 85${}^{\circ }$, 75${}^{\circ }$ and 60${}^{\circ }$ compared to the other ones can be explained by the missing averaging between cycle 1 and cycle 2.

## 5. Conclusions and Outlook

To increase the accuracy of geodetic and astronometric measurements performed by a radio telescope, its elevation-dependent deformation should be known very accurately. Therefore, the present study presents a new measurement and data-processing concept based on a terrestrial laser scanner, applied to the OSO 20-m radio telescope. In total, three main aspects are handled:

- Scanning in two faces
  A new measurement concept for deformation analyses of radio telescopes’ main reflectors is proposed based on scanning in two faces to minimize systematic measurement errors due to misalignment of the TLS.
- Accuracy evaluation of TLS measurements
  The accuracy of the laser scans is evaluated by analyzing the stability of the laser scanner and by assessing the precision of the measured distances. Special focus is led onto the systematic measurement errors due to misalignment of the TLS. These preliminary investigations prove the applicability of laser scanners for the present task.
- Elevation-dependent deformation analysis
  For the deformation analysis, a new concept is presented including the two-faces measurements: It is based on an in situ calibration of the laser scanner in a bundle adjustment including all elevation angles.

Concerning the OSO 20-m radio telescope, focal length variations of 9.6 mm between the elevation angles of 85${}^{\circ }$ to 5${}^{\circ }$ can be determined. This value is quite large compared to the 100-m Effelsberg radio telescope whose focal length decreases by 22.7 mm [18]. Further local deformations of the main reflector cannot be detected. The estimated residuals can only be assigned to the laser scanners measurement accuracy.

In the next steps of data analysis, some general aspects should be considered further: Firstly, the results should be used to improve the VLBI delay model leading to more accurate baseline estimates. First considerations can be found in [43]. Secondly, while the deformation parameters are attested to be significant based on metrological expertise, performing a congruence test as usual at deformation analyses [44] is not productive since the stochastic model of laser scans is hardly known so far. The determination of a more realistic stochastic model of laser scans should, therefore, be focused on in prospective studies. Thirdly, further strategies for dealing with systematic errors due to the misalignment of the TLS at deformation analyses should be discussed.

## Figures and Tables

**Table 1:** Estimated calibration parameters ${\hat{p}}_{calib}$.

Value	${\hat{x}}_{1z}$ (mm)	${\hat{x}}_{3}$ (mm)	${\hat{x}}_{5z-7}$ (”)	${\hat{x}}_{6}$ (”)	${\hat{x}}_{1n+2}$ (mm)	${\hat{x}}_{4}$ (”)	${\hat{x}}_{5n}$ (”)
${\hat{p}}_{calib}$	−0.2	−0.3	92.6	5.3	−0.9	17.1	10.5
${\hat{\sigma }}_{calib}$	0.02	0.02	0.39	0.20	0.04	0.09	0.78

**Table 2:** Estimated object parameters ${\hat{p}}_{obj}$.

Elevation Angle	${\hat{X}}_{v}$ (m)	${\hat{Y}}_{v}$ (m)	${\hat{Z}}_{v}$ (m)	${\hat{\phi }}_{x}$ (${}^{\circ }$)	${\hat{\phi }}_{y}$ (${}^{\circ }$)	$\hat{f}$ (m)	${\hat{\sigma }}_{f}$ (mm)	$\Delta \hat{f}$ (mm)
85${}^{\circ }$	−2.0923	−0.6992	7.0804	182.8879	5.4603	8.9910	0.05	–
75${}^{\circ }$	−1.9574	−0.9244	7.0976	186.5480	15.3586	8.9889	0.05	−2.1
60${}^{\circ }$	−1.7832	−1.0990	7.1434	192.0874	30.4292	8.9900	0.05	−1.0
45${}^{\circ }$	−1.7907	−0.9740	7.2152	201.7932	44.0489	8.9879	0.03	−3.1
30${}^{\circ }$	−1.8772	−0.7155	7.2994	214.2344	55.3794	8.9856	0.03	−5.4
15${}^{\circ }$	−1.9881	−0.0186	7.4017	239.2063	63.6801	8.9837	0.03	−7.3
5${}^{\circ }$	−1.8869	0.6217	7.4709	260.8560	65.5353	8.9814	0.03	−9.6

## Abbreviations

The following abbreviations are used in this manuscript:

GHM	Gauß-Helmert Model
ICP	Iterative Closest Point Algorithm
OSO	Onsala Space Observatorium
TLS	Terrestrial Laser Scanner
VLBI	Very Long Baseline Interferometry

## References

[1] Ioannidis C., Valani A., Georgopoulos A., Tsiligiris E. 3D model generation for deformation analysis using laser scanning data of a cooling tower. Proceedings of the 3rd IAG/12th FIG Symposium.

[2] Van Gosliga R., Lindenbergh R., Pfeifer N. (2006). Deformation analysis of a bored tunnel by means of terrestrial laser scanning. Int. Arch. Photogramm. Remote Sens. Spat. Inf. Sci..

[3] Eling D. (2009). Terrestrisches Laserscanning für die Bauwerksüberwachung. Ph.D. Thesis.

[4] Wang J. (2013). Towards Deformation Monitoring with Terrestrial Laser Scanning Based on External Calibration and Feature Matching Methods. Ph.D. Thesis.

[5] Filipiak-Kowszyk D., Janowski A., Kaminski W., Makowska K., Szulwic J., Wilde K. (2016). The Geodetic Monitoring of the Engineering Structure—A Practical Solution of the Problem in 3D Space. Rep. Geod. Geoinform..

[6] Holst C., Neuner H., Wieser A., Wunderlich T., Kuhlmann H. (2016). Calibration of Terrestrial Laser Scanners. Allgem. Verm. Nachr..

[7] Lichti D., Chow J., Lahamy H. (2011). Parameter de-correlation and model-identification in hybrid-style terrestrial laser scanner self-calibration. ISPRS J. Photogramm..

[8] Lichti D. (2010). Terrestrial laser scanner self-calibration: Correlation sources and their mitigation. ISPRS J. Photogramm..

[9] Holst C., Kuhlmann H. (2014). Aiming at self-calibration of terrestrial laser scanners using only one single object and one single scan. J. Appl. Geod..

[10] Abbas M.A., Lichti D.D., Chong A.K., Setan H., Majid Z., Lau C.L., Idris K.M., Ariff M.F.M. (2017). Improvements to the accuracy of prototype ship models measurement method using terrestrial laser scanner. Measurement.

[11] Muralikrishnan B., Ferrucci M., Sawyer D., Gerner G., Lee V., Blackburn C., Phillips S., Petrov P., Yakovlev Y., Astrelin A. (2015). Volumetric performance evaluation of a laser scanner based on geometric error model. Precis. Eng..

[12] Muralikrishnan B., Shilling K.M., Sawyer D.S., Rachakonda P.K., Lee V.D., Phillips S.D., Cheok G.S., Saidi K.S. Laser scanner two-face errors on spherical targets. Proceedings of the Annual Meeting of the ASPE 2014.

[13] Medić T., Holst C., Kuhlmann H. (2017). Towards system calibration of panoramic laser scanners from a single station. Sensors.

[14] Dutescu E., Heunecke O., Krack K. (2009). Formbestimmung bei Radioteleskopen mittels Terrestrischem Laserscanning. Allgem. Verm. Nachr..

[15] Sarti P., Vittuari L., Abbondanza C. (2009). Laser scanner and terrestrial surveying applied to gravitational deformation monitoring of large VLBI telescopes’ primary reflector. J. Surv. Eng..

[16] Sarti P., Abbondanza C., Vittuari L. (2009). Gravity-dependent signal path variation in a large VLBI telescope modelled with a combination of surveying methods. J. Geod..

[17] Holst C., Zeimetz P., Nothnagel A., Schauerte W., Kuhlmann H. (2012). Estimation of focal length variations of a 100-m radio telescope’s main reflector by laser scanner measurements. J. Surv. Eng..

[18] Holst C., Nothnagel A., Blome M., Becker P., Eichborn M., Kuhlmann H. (2015). Improved area-based deformation analysis of a radio telescope’s main reflector based on terrestrial laser scanning. J. Appl. Geod..

[19] Holst C., Kuhlmann H. (2016). Challenges and Present Fields of Action at Laser Scanner Based Deformation Analyses. J. Appl. Geod..

[20] Johansson L.E., Olofsson A., Beck E.D. (2016). OSO 20 m Telescope Handbook.

[21] Clark T.A., Thomsen P. (1988). Deformations in VLBI Antennas.

[22] Sarti P., Abbondanza C., Petrov L., Negusini M. (2011). Height bias and scale effect induced by antenna gravitational deformations in geodetic VLBI analysis. J. Geod..

[23] Soudarissanane S., Lindenbergh R., Menenti M., Teunissen P. (2011). Scanning geometry: Influencing factor on the quality of terrestrial laser scanning points. ISPRS J. Photogramm..

[24] Zámečniková M., Neuner H. (2017). Untersuchung des gemeinsamen Einflusses des Auftreffwinkels und der Oberflächenrauheit auf die reflektorlose Distanzmesssung beim Scanning. Ingenieurvermessung 17: Beiträge zum 18. Internationalen Ingenieurvermessungskurs.

[25] Wujanz D., Burger M., Mettenleiter M., Neitzel F. (2017). An intensity-based stochastic model for terrestrial laser scanners. ISPRS J. Photogram..

[26] Lichti D. (2007). Error modelling, calibration and analysis of an AM-CW terrestrial laser scanner system. ISPRS J. Photogramm..

[27] Chow J., Lichti D., Glennie C. (2011). Point-based versus plane-based self-calibration of static terrestrial laser scanners. Int. Arch. Photogramm. Remote Sens. Spat. Inf. Sci..

[28] Chow J., Lichti D., Glennie C., Hartzell P. (2013). Improvements to and comparison of static terrestrial lidar self-calibration methods. Sensors.

[29] Lichti D., Gordon S.J., Tipdecho T. (2005). Error models and propagation in directly georeferenced terrestrial laser scanner networks. J. Surv. Eng..

[30] Lichti D. (2009). The impact of angle parameterisation on terrestrial laser scanner self-calibration. Int. Arch. Photogramm. Remote Sens. Spat. Inf. Sci..

[31] Zainuddin K., Setan H., Majid Z. (2009). From laser point cloud to surface: data reduction procedure test. Geoinform. Sci. J..

[32] Lee K., Woo H., Suk T. (2001). Data reduction methods for reverse engineering. Int. J. Adv. Manuf. Technol..

[33] Lee K., Woo H., Suk T. (2001). Point data reduction using 3D grids. Int. J. Adv. Manuf. Technol..

[34] Holst C., Artz T., Kuhlmann H. (2014). Biased and unbiased estimates based on laser scans of surfaces with unknown deformations. J. Appl. Geod..

[35] Roscher R. (2013). Sequential Learning Using Incremental Import Vector Machines for Semantic Segmentation. Ph.D. Thesis.

[36] Weiss U., Biber P. (2011). Plant detection and mapping for agricultural robots using a 3D lidar sensor. Robot. Auton. Syst..

[37] Leica Geosystems Leica ScanStation P20, Industry’s Best Performing Ultra-High Speed Scanner. www.leica-geosystems.de.

[38] Jurek T., Kuhlmann H., Holst C. (2017). Impact of spatial correlations on the surface estimation based on terrestrial laser scanning. J. Appl. Geod..

[39] Kauker S., Schwieger V. (2017). A synthetic covariance matrix for monitoring by terrestrial laser scanning. J. Appl. Geod..

[40] Mikhail E., Ackermann F. (1976). Observations and Least Squares.

[41] Wolf H. (1975). Ausgleichungsrechnung. Formeln zur Praktischen Anwendung.

[42] Neitzel F. (2010). Generalization of total least-squares on example of unweighted and weighted 2D similarity transformation. J. Geod..

[43] Nothnagel A., Holst C., Schunck D., Haas R., Wennerbäck L., Olofsson H., Hammargren R. Paraboloid deformation investigations of the Onsala 20 m radio telescope with terrestrial laser scanning. Proceedings of the 23rd Meeting of the European VLBI Group for Geodesy and Astronomy.

[44] Heunecke O., Kuhlmann H., Welsch W., Eichhorn A., Neuner H. (2013). Hanbuch Ingenieurgeodäsie. Auswertung Geodätischer Überwachungsmessungen.

